# Different perceptions of narrative medicine between Western and Chinese medicine students

**DOI:** 10.1186/s12909-017-0925-0

**Published:** 2017-05-10

**Authors:** Chien-Da Huang, Kuo-Chen Liao, Fu-Tsai Chung, Hsu-Min Tseng, Ji-Tseng Fang, Shu-Chung Lii, Han-Pin Kuo, San-Jou Yeh, Shih-Tseng Lee

**Affiliations:** 1 Chang Gung Medical Education Research Center, Chang Gung Memorial Hospital, Chang Gung University, College of Medicine, 199 Tun Hua N. Rd, Taipei, Taiwan; 2 Department of Medical Education, Chang Gung Memorial Hospital, Chang Gung University, College of Medicine, Taipei, Taiwan; 3 Thoracic Medicine, Chang Gung Memorial Hospital, Chang Gung University, College of Medicine, Taipei, Taiwan; 4 General Medicine, Chang Gung Memorial Hospital, Chang Gung University, College of Medicine, Taipei, Taiwan; 5 Nephrology, Chang Gung Memorial Hospital, Chang Gung University, College of Medicine, Taipei, Taiwan; 6 Cardiology, Chang Gung Memorial Hospital, Chang Gung University, College of Medicine, Taipei, Taiwan; 7 Neurosurgery, Chang Gung Memorial Hospital, Chang Gung University, College of Medicine, Taipei, Taiwan; 8 Department of Health Care Management, Chang Gung University, College of Medicine, Taipei, Taiwan; 9 Department of Medical Humanities and Social Sciences, Chang Gung University, College of Medicine, Taipei, Taiwan

**Keywords:** Narrative medicine, Perception, Medical students, Chinese medicine, Learning culture

## Abstract

**Background:**

Western medicine is an evidence-based science, whereas Chinese medicine is more of a healing art. To date, there has been no research that has examined whether students of Western and Chinese medicine differentially engage in, or benefit from, educational activities for narrative medicine. This study fills a gap in current literature with the aim of evaluating and comparing Western and Chinese Medicine students’ perceptions of narrative medicine as an approach to learning empathy and professionalism.

**Methods:**

An initial 10-item questionnaire with a 5-point Likert scale was developed to assess fifth-year Western medical (MS) and traditional Chinese medical (TCMS) students’ perceptions of a 4-activity narrative medicine program during a 13-week internal medicine clerkship. Exploratory factor analysis was undertaken.

**Results:**

The response rate was 88.6% (412/465), including 270 (65.5%) MSs and 142 (34.5%) TCMSs, with a large reliability (Cronbach alpha = 0.934). Three factors were extracted from 9 items: personal attitude, self-development/reflection, and emotional benefit, more favorable in terms of enhancement of self-development/reflection. The perceptions of narrative medicine by scores between the two groups were significantly higher in TCMSs than MSs in all 9-item questionnaire and 3 extracted factors.

**Conclusions:**

Given the different learning cultures of medical education in which these student groups engage, this suggests that undertaking a course in Chinese medicine might enhance one’s acceptance to, and benefit from, a medical humanities course. Alternatively, Chinese medicine programmes might attract more humanities-focused students.

## Background

Following advances in medical informatics and technology, nowadays medicine *as science for practicing* is much easier than *medicine as an art for healing* [1, 2]. However, medicine is not merely the taking of a comprehensive history, physical examination, diagnostic workup and the final discussion about a plan for action [1, 3]. Although some academics have contested the universality of the notion, [4, 5] many argue that medicine requires physicians to establish narrative connections and dive deep into the crying soul of the patient [1, 6].

### Narrative medicine

Rita Charon first used the phrase “narrative medicine” in 2000 to refer to clinical practice fortified by narrative competence [7]. Narrative medicine is medicine performed with narrative skill and offered as a model for humanism and effective medical practice, illuminating medicine’s crucial narrative relations: physician and patient, physician and self, physician and colleagues, and physicians and society [6, 8]. Such narrative skill can be developed through engaging in one’s own narrative reading and writing episodes, particularly through expressive writing: “Building a narrative, then, may be critical in reaching understanding or knowledge” [9]. The development of narrative skill can also include listening to stories of patients, self- reflection, and reflection on the profession as a whole, or even on society as a whole [8, 10].

For physicians, narrative medicine is therefore thought to be a good way to understand the personal connections between themselves and their patients [6]. It is believed to help physicians recognize, interpret, and be moved to action by the problems of others and develop confidence and competence while identifying the conflicts they face [6]. Narrative medicine practitioners assert that physicians are able to link to their patients with narrative competence; further understanding their own personal journeys through medicine; recognise empathy with, and responsibilities toward, other health care professionals; and engaging in meaningful discussions with the public about health care [11]. By connecting physicians to patients, colleagues and society, narrative medicine is thought to provide new opportunities for respectful, empathic, and nourishing medical care [12–14].

### Medical students’ reactions to narrative medicine

Research suggests that there is a benefit for physicians-in-training and surgical residents to engage patients, to elicit their story in the context of illness and to undertake reflection through narrative writing [15, 16]. Furthermore, medical students believe narrative medicine to be an important, effective, and counter-cultural means of enhancing their communication, collaboration, and professional development by supporting complex interior, interpersonal, perceptual and expressive capacities [17, 18]. Indeed, an experiential narrative medicine curriculum for medical students involving patient storytelling and group reflection was found to be feasible and acceptable to both patients and students, with some patients and students being profoundly moved by the experience [19]. However, studies examining the impact of narrative medicine training have predominantly been conducted within a Western medicine perspective, even when the research context itself comprises a non-Western setting [20]. Furthermore, even research that includes a mixture of students studying Western and Chinese medicine has failed to examine differences between these two groups [21]. As such we do not know whether the concept of narrative medicine will be equally well received across these different medical cultural contexts.

### Differences between Western and traditional Chinese medicine

Western medicine uses a reductive and analytical approach, while Chinese medicine uses an inductive and synthetic approach. Chinese medicine, however, lacks proper diagnostic tools, whereas Western medicines’ strength is its powerful diagnostic ability [22]. Thus, Western medicine is based on standards and evidence, with Chinese medicine relying on experience over time through numerous trials and clinical observations [22, 23]. In short, while Western medicine is a standardized and evidence-based science, Chinese medicine is experience-based and more of a healing art [22].

### Traditional features of Chinese medicine

The ancient medical scientists within traditional Chinese medicine explain the phenomenology of human physiology, pathology-based Yin and Yang and summarize the medical experience. Chinese medicine has taken the human body as a whole, from the key concepts of “qi, blood, yin-yang, viscera (Zang-Fu), and meridian and channel”, rather than a single cell or a particular organ [23]. In Chinese medicine, disease is not viewed as something that a patient has, but something that the patient is. Disease is considered as an imbalance in the patient’s being. There is only a whole person whose body functions may be balanced or imbalanced, harmonious or disharmonious. Thus, understanding the nature of the imbalance is the goal of diagnosis, while restoring balance is the focus of treatment [22]. This suggests that people studying Chinese medicine might therefore have a different world-view than those studying medicine within a Western framework, and this world-view might impact on how they engage with different aspects of their curricular, including narrative medicine activities.

This study aims to fill the gap in current literature by comparing Western and Chinese Medicine students’ perceptions of narrative medicine as an approach to learning empathy and professionalism. We specifically asked the following research question: Are there any differences in the perception of a narrative medicine programme between Western and Chinese Medicine students during a pre-doctor clerkship?

## Methods

### Context

The context of this study is a large teaching hospital in Taiwan. Western medical students (MS) have a 7-year undergraduate programme [24]. During the first 2 years, they not only have fundamental courses that provide an introduction to medicine, but they also have courses related to general education, such as information technology, art and literature. In the following 2 years, they have the opportunity to learn further medical skills and knowledge. From Year 5, students have an opportunity to apply the knowledge they have gained in practice: each student completes a 2-year clerkship and a 1-year internship in hospitals so they can master all the required techniques and know-how of patient care.

Traditional Chinese medical students (TCMS) have an 8-year undergraduate programme. The characteristic of the Department of Traditional Chinese Medicine is the teaching of both Chinese and Western medicine. The courses offered include Basic Education, General Education, Basic Education of Chinese and Western Medicine, Clinical Education of Chinese and Western Medicine, Practice of Western Medicine (7th year), and Practice of Chinese Medicine (8th year). In order to develop the students’ life-long learning ability, problem oriented teaching is added to the courses in coordination with the General Education. This course focuses on the nurture of personalities with the aim of developing excellent doctors with ability and moral integrity in a combination of both Chinese and Western medicine. Thus, when they are in a practice environment, they are more aware of the health attitudes of their patients from different ethnic backgrounds, leading to a better patient-doctor relationship and better adherence with treatment [25].

The narrative medicine model was introduced into the clerkship program of 5th-year clerks, for both MSs and TCMSs. Using a narrative approach, clerks write about daily clinical events and encounters, their struggles and their accomplishments without the critical eye of the preceptor, the attending or their seniors.

#### The narrative medicine program activities

*Activity 1:* The protocol for narrative writing begins with a typical lecture explaining the theory and introducing the process. This activity is integrated as a one-hour session into the curriculum of Basic Clinical Skills for Medical Clerkship.

*Activity 2:* A narrative medicine workshop for clinical teachers.

This workshop is held twice each year – BEGAN (The Brown Educational Guide to the Analysis of Narrative) [26] – A framework for enhancing educational impact of faculty feedback to students’ reflective writing is used.

*Activity 3:* In the session, medical students are allowed to represent clinical stories in their narrative writing assignments in different ways, such as story-telling or poetry-reading. This activity is designed to enhance humanism sensitivity of medical students through the processes of enabling medical students to recognize, to interpret and to be moved to action by the problems of others. Through the act of narrative writing, medical students can review their journeys through their clerkship: rethinking and reflecting on the stories they gather from patients. One of the objectives of this course is the opportunity for medical students to transfer their cognitive knowledge and attitudes of physician-patient communication into practice thus facilitating a better understanding of patients.

*Activity 4:* This comprises a small group discussion between 6 and 8 medical students and one clinical teacher facilitator. In this one-hour activity, each student reads his or her narrative writing assignment, reflects on the experiences of their patient encounter and receives feedback from peers and the facilitator. The performance of each student is assessed by the modified REFLECT (Reflection Evaluation For Learners’ Enhanced Competencies Tool) Rubric [27].

Four hundred and sixty five medical students compulsorily participated in this 4-activity narrative medicine program during a 13-week clerkship of internal medicine during the 2012–2014 academic years.

### Study methods and participants

A three-cohort cross-sectional questionnaire study design was used. The questionnaire comprised a 10-item survey instrument administered using a 5-point Likert scale (strongly disagree to strongly agree). Ethical approval was obtained from the Chang Gung Memorial Hospital and Chang Gung University Institutional Review Boards (IRB No. 102-4138B, No.103-1755B, 105-2716C, 106-1287C) and participation in the study was voluntary.

#### Questionnaire development

The survey instrument investigated two domains of student perceptions: perceptions about the narrative medicine activity and personal attitudes about the narrative medicine progress model. Perceptions about the narrative medicine activity included items such as “Narrative medicine (NM) is helpful for reflection, enhancement of empathy, relationship between patients and doctors, and relieving my grief during medical care”, “NM is essential for medical care” and “NM relieves my pressure during medical care”. While, personal attitudes about the narrative medicine progress model included “I have a good overall impression on NM”, “I am interested in NM”, “I will tell my junior schoolmates about the concept of NM” and “I will continue with my narrative writing”. Four experts in clinical education and faculty development reviewed the items for content and content validity. A pilot check with faculty members was performed examining internal consistency and reliability.

#### Procedure

Students were invited to participate in the study anonymously at the end of each semester. The questionnaire was voluntary and open for a 14-day period. A total of 412 fifth year medical students completed the questionnaires (412/465 = 88.6%), including 270 (65.5%) MSs and 142 (34.5%) TCMSs: 255 (61.9%) were male and 157 (38.1%) were female students. There were 123 (29.9%), 159 (38.6%), and 130 (31.6%) in 2012, 2013, and 2014 academic years respectively. There was no cohort difference.

#### Exploratory factor analysis

An exploratory factor analysis (EFA) was conducted using principal component analysis to test the assumption of whether the questionnaire contained two underlying factors as originally designed. The Kaiser–Meyer–Olkin measure of sampling adequacy was 0.939, and Bartlett’s test of sphericity was significant (*p* < 0.0001), indicating that the sample was suitable for factor analysis. Factors were extracted and rotated to orthogonal simple structure using the varimax method.

In the following section, data are expressed as mean values and standard deviation (SD) or as numeric values (%). Unpaired *t*-tests were used to compare 5-Likert scales that were considered to be parametric data. The level of statistical significance was set at *p* < 0.05. All analyses were conducted using SPSS software (version 13.0, SPSS, Chicago, IL).

## Results

### Three factors identified: Personal attitude, self-development/reflection, emotional benefit

Several criteria were taken into account simultaneously to determine how many factors to settle on. First, we used the Kaiser’s criterion and the scree plot which showed that three of those factors explain most of the variability because the line starts to straighten after factor 3. The remaining factors explained a very small proportion of the variability (accounting for 19.7%) and thus likely unimportant. Secondly and most importantly, we wanted to get a reasonable proportion of variance explained and substantive sense of the data achieved. The results suggested that the first three factors, accounting for 80.3% of the total variance, should be retained (Table 1). Several questions (Item 3, Item 4, and Item 5) had their highest loading from the first factor but had a cross-loading over 0.4 on the competence factor. In order to deal with the cross loadings issue, we examined the factor loadings using oblimin to see if these cross-loadings still appeared. The results suggested that Item 5 continued to load on multiple factors after using the oblimin rotation. Therefore, this item was discarded.

**Table 1:** Exploratory factor analysis: rotated component matrix

Items		Factors		
		Personal attitude	Self-Development/Reflection	Emotional benefit
1	I will continue with my narrative writing	.846	.238	.269
2	I will tell my junior schoolmates about the concept of NM	.818	.285	.292
3	I have a good overall impression on NM	.727	.440	.270
4	I am interested in NM	.688	.385	.405
5	NM is essential for medical care	.550	.427	.532
6	NM is helpful for reflection	.314	.811	.162
7	NM is helpful for in enhancing empathy	.295	.766	.282
8	NM is helpful for patients-doctor relationships	.295	.737	.396
9	NM relieves my pressure during medical care	.324	.228	.842
10	NM relieves my grief during medical care	.314	.335	.777

*NM* narrative medicine

On the basis of the characteristics of the items that constituted each factor, the first factor was interpreted as “personal attitude” (4 items), the second as “self-development/reflection” (3 items) and the third as “emotional benefit” (2 items). Students’ responses to this 9-item questionnaire yielded a Cronbach alpha measure of reliability equal to 0.934. Cronbach’s alpha coefficients for each subscale were 0.92, 0.85, and 0.72 respectively.

For the three-factor model, the mean and standard deviation scores of subscales personal attitude, self-development/reflection, and emotional benefit were 3.35 ± 0.995, 3.82 ± 0.871, and 3.19 ± 1.056 respectively.

### Western versus Chinese medical student differences

The unique aspect of this project is that we compared and analyzed responses from two different groups: Western (MSs) and Chinese (TCMSs) medicine. Narrative medicine scores were significantly higher in TCMSs than MSs for all 9 items. They were also significantly higher in TCMSs than MSs for all three factors: personal attitude (*p* < 0.001), self-development/reflection (*p* < 0.001) and emotional benefit (*p* < 0.01). TCMSs had better global perception for total factors than MSs (*p* < 0.001) (Table 2). There was no difference in terms of gender on the perceptions on narrative medicine for all 9 items, although there were more females in the TCMS group.

**Table 2:** Comparison of perception towards narrative medicine between students of Medicine department and Chinese Medicine department

Factors	Department	*N*	Mean	SD
Personal attitude (4 items, Item 1–4)	MS	270	3.26	1.028
	TCMS	142	3.52	0.906
Self-development/Reflection (3 items, Item 6–8)	MS	270	3.74	0.908
	TCMS	142	3.98	0.774
Emotional benefit (2 items, Item 9–10)	MS	270	3.10	1.061
	TCMS	142	3.34	1.033
Total (9 items)	MS	270	3.39	1.030
	TCMS	142	3.63	0.930

*MS* medical student, *TCMS* traditional Chinese medical student

## Discussion

This study aimed to assess the impact of narrative medicine for 5th year Western and Chinese medical students using a purposively developed questionnaire. Three factors were extracted from the 9 questionnaire items: personal attitude, self-development/reflection and emotional benefit. Overall, students were significantly more likely to agree that the course facilitated their self-development and reflection than it facilitating personal attitudes about narrative medicine and emotional benefits. This finding that students’ empathy, reflection and interpersonal relationships all benefit from their engagement with the narrative medicine course resonates with previous research analysing writing content during courses. Through these narratives students described how their engagement in narrative writing, they received a greater understanding of themselves, patients’ and families’ feelings, reflection, humanistic situations, and motivation to improve [12–14, 16].

The current study is unique in that we examined two different groups of medical students: Western (MSs) and Chinese (TCMSs) medicine students. We found perceptions of narrative medicine to be significantly higher in TCMSs than MSs for each of the 9 items in the questionnaire and therefore for the 3 extracted factors. Such a finding leads us to wonder why this might be so. As discussed above, the characteristics of traditional Chinese medicine in both theory and practice make it very different from conventional Western Medicine [28]. A distinctive difference between the two is that Western medicine focuses solely on the body as an organism. On the other hand, Chinese medicine considers the soul or spirit as an integral element of the body. Therefore, one essential difference between them is that through the learning of Chinese medicine, students are trained to conceptualise the entire body in an holistic manner to solve a single ailment, rather than focusing on disparate body parts (which is more of a feature of Western medicine). Furthermore, Chinese medicine not only offers professional medical knowledge, but it also emphasises humanities, social, legal, and ethical education [22, 23]. Having said this, there might be other alternative explanations, including that Chinese medicine might attract more humanities-focused students.

We believe that role modeling may also partially explain this difference. Role modeling is thought to be an integral component of medical education and an important factor in shaping the values, attitudes, behavior, and ethics of medical trainees. In comparison with traditional Chinese medicine, Western medicine strongly features scientific technology, with frequent use of high-technology instruments as a defining feature: while not an exact science, behind many diagnoses lie a variety of measurements with developed formula for signs of specific pathology. Thus the focus is split between measurements and the patient, with emotional detachment from the patient being a defining feature of professionalism. Traditional Chinese medicine doctors operate differently. They typically communicate with their patients, focusing on patients’ feelings during diagnoses, taking the human body as balance and harmony. Therefore, although the symptoms might be the same, Chinese and Western medicine physicians have very different relationships with patients and offer different therapies based on their personal experiences [29]. Thus, traditional Chinese medicine physicians as clinical teachers and therefore role models, demonstrate how to focus on patients’ illness through caring for their feelings. Given that the presence of role models during clinical training is a determining factor in the acquisition of medical expertise, including enhancing learning, influencing career choices and facilitating the acquisition of humanistic attitudes [30, 31], we believe this powerful force is partly the reason why we find such a difference between student groups: TCMSs being more familiar with the concept of medicine as an holistic healing art. This state of affairs not only plays into students’ recognition, and therefore acceptance of narrative medicine, but it also impacts on students’ willingness to go the extra mile: our narrative medicine programme relied on the clerks committing their own time to participate. Thus the different learning cultures of MS and TCMS medical education affect both acceptance to, and benefit from, a narrative medicine course.

The predominance of female students in medical education is an important issue. A four-nation study published in 2002 conducted in Western cultures (Australia, Canada, England and the United States) showed that women make up half of all medical students and 30% of all practicing physicians [32]. In many Western cultures, like the United Kingdom, women even form the majority of the physician workforce [33]. However, female doctors are a relative minority in Taiwan. By 1980, Taiwan had only around 4.3% female doctors. The percentage has steadily increased: from 6% in 1990 to 12% in 2000. Female medical graduates have also increased from 1990 (10%) to 2000 (29.9%) [34]. There are 29.1% female medical students in our previous study in 2013, in which females had better behavior records and more attendance in community services than men [35]. Furthermore, research suggests that Tunisian female students attach more value than males to the intrinsic aspects of a physician’s job, such as the desire to help others and to work with people [36]. In a psychometric study of candidates admitted to Scottish medical schools, female applicants as a group were identified as being more empathic, with a greater communitarian orientation than men [37]. Patient-Practitioner Orientation Scale (PPOS) scores from Swedish female students were higher compared to their male counterparts, and females scored significantly higher in later in their studies compared with early on [38]. However, in our study, we have found no difference in terms of gender on the perceptions on narrative medicine, despite there being more females in TCMS. This suggests that the learning culture between MS and TCMS is the predominant factor rather than that of gender.

### Strengths and weaknesses of the study

This study, recruiting clerks from different training programs (MSs and TCMSs), obtained a high response rate and analyzed data collected at the end of a 13-week long study employing prompted narrative medicine. The current study is unique, being the first to examine the perceptions of narrative medicine between two different groups of medical students: MSs and TCMSs.

There are some limitations to this study. First, the study was surveyed in an internal medicine rotation only, but not in pediatrics, or surgical systems such as surgery or gynecology. Although this work was carried out in two separate groups of clerks, caution should be taken in generalizing the findings to other medical trainees apart from the Department of Traditional Chinese Medical. Secondly, our MSs or TCMSs followed a prospective-based program on clerkship narrative medicine, however they could not be randomized. For the purposes of data analysis, we did not hypothesize that their choice of specialization would be a significant contributing factor. Nevertheless, this is unlikely to be an important factor as the students were from two different groups. Thirdly, as previously mentioned, there is the possibility that the two programs of medical education may attract different kinds of students: Chinese medicine might seem more suitable for holistic thinkers who could be more open to the humanities. As such this could be a possible contributor to the differential impact of the narrative medicine course. Finally, narrative medicine as a teaching practice has been criticized as being too naïve a use of ‘reflective practice’ in pedagogy [39, 40]. Indeed, the key issues that underpin how such practices can be valuable are far more complex than could be captured in this single study. Thus further studies are needed to better understand the differences between MS and TCMS groups of students in their engagement with, and reactions towards, narrative medicine.

### Suggestions for practice

The act of narrative writing involves thinking explicitly about a thought, experience, or action and has profound implications for medical education [41, 42]. Thus, educators may consider incorporating narrative writing into their curriculum to promote humanism since it may be difficult for young pre-doctors to feel, understand, and think about humanistic situations. For medical students, narrative medicine may be a good complement to the philosophies of a bio-psychosocial approach and patient-centered practice and could be brought in at an earlier point in the curriculum for Western medicine students as an ‘antidote’.

The most important aspect of narrative writing is to review one’s assumptions or beliefs to enhance self-awareness. A medical student’s journey through the healthcare system is often difficult and accompanied with self-doubts and frustration. Although we have not found our narrative medicine course to be best at relieving their own grief or pressure, our hope is that by having the clerks begin writing about their experiences, they will gain a sense of themselves through reflection over time and, through the sharing of stories, see that others feel the same or similar to themselves.

### Suggestions for future research

In this study, three factors were extracted: personal attitude, self-development/reflection, and emotional benefit. We also found that the perceptions of narrative medicine were significantly higher in the TCMS than the MS group. However, definitions and actualisations of narrative or narrative medicine are often broad. Indeed, what lies within the ‘black box’ of narrative medicine needs to be better explored [43–45]. Despite the different learning cultures of medical education in which these student groups engage can partially explain our results, we do not fully understand what it is about narrative medicine that works, for whom, and why across different learning culture contexts, As such, the impact of learning cultures deserves to be further studied in order to explore the complex interactions and to develop a transferable theoretical model of what works, for whom and why [46]. In this way, not only will we understand similarities and differences between Chinese and Western medical students learning with narrative medicine processes, we will also be able to tailor educational strategies to promote narrative medicine into early pre-doctoral medical education for specific groups and further enhance reflection and humanism.

## Conclusions

In our study, students from both TCMS and MS groups perceived their narrative medicine learning experiences to be potentially beneficial to their self-development and capacity for reflection. The perceptions of the narrative medicine course, however, were more favorable in the TCMS group than in the MS group. The characteristics of TCM in both theory and practice make it different from conventional Western Medicine. Given the different learning cultures of medical education in which these groups engage, it could be that undertaking a course in Chinese medicine might actually enhance one’s acceptance to, and benefit from, a medical humanities course. Alternatively, Chinese medicine might attract more humanities-focused students.

## Abbreviation

BEGAN: The Brown Educational Guide to the Analysis of Narrative
EFA: exploratory factor analysis
MS: medical student
NM: narrative medicine
REFLECT: Reflection Evaluation For Learners’ Enhanced Competencies Tool
SD: standard deviation
TCMS: traditional Chinese medical student
